# Whole-mount immunostaining that avoids cross-reaction between antibodies from different host species for simultaneous visualization of actin filaments and microtubules

**DOI:** 10.5511/plantbiotechnology.24.1103a

**Published:** 2025-03-25

**Authors:** Toshiki Amari, Natsu Higashinaka, Masaki Ito, Hirotomo Takatsuka

**Affiliations:** 1 School of Biological Science and Technology, College of Science and Engineering, Kanazawa University, Kakuma-machi, Kanazawa, Ishikawa 920-1192, Japan

**Keywords:** actin, cytoskeleton, immunostaining, microtubule, root

## Abstract

The plant cytoskeleton, composed of microtubules and actin filaments, is an essential structural element for plant growth and development; it optimizes cell size and shape along the differentiation trajectories. Thus, visualizing and observing the cytoskeleton’s spatial organization within cells is crucial to better understanding plants’ developmental strategies as sessile organisms. Here, we developed a whole-mount immunostaining method for double-labeling actin filaments and microtubules using *Arabidopsis thaliana* roots. To enable this, we examined the specificity of the secondary antibody toward the primary antibody raised in different host-species to propose two optimal methods to double-label actin filaments and microtubules, depending on the combinations of the host-species for primary antibodies: “simultaneous immunostaining”, in which two sets of primary and secondary antibodies are applied simultaneously and “sequential immunostaining”, where two rounds of antibody-antigen reactions are conducted sequentially. The sequential reaction aims to avoid cross-species immunoreaction, where the secondary antibody undesirably binds to the primary antibody from a different host species. Our findings can provide valuable information on how to select antibodies not only for the cytoskeletal elements but also for other proteins of interest.

The plant cytoskeletal system comprises microtubules (MTs) and actin filaments (AFs). The α- and β-tubulin heterodimers polymerize into MTs, while AFs include monomeric globular proteins called G-actin (Hohmann and Dehghani 2019; Kost and Chua 2002). The plant cytoskeleton is crucial in determining cell shape and growth direction. For example, longitudinally elongating root and hypocotyl cells bear transversely aligned MTs at their cortex that guide the deposition of cellulose microfibrils in the same direction (Paredez et al. 2006). The resulting cellulose microfibrils prevent the cells from swelling, guaranteeing cell growth in the longitudinal direction. In contrast, just before the onset of root cells’ active growth, fine and meshed AFs are reorganized into thick, longitudinally oriented bundles, thus driving cell growth in a longitudinal direction (Takatsuka et al. 2018). MTs and AFs have been regarded as structural elements that determine cell size and shape differently. Given their importance, visualizing the cytoskeletons and observing their organization and distribution is essential to better understand the nature of plant cells.

Advances in fluorescent proteins (FPs) have greatly improved our understanding of how cytoskeletons behave within the cell. Nonetheless, due to technical limitations, employing FPs alone cannot fully uncover how AFs and MTs are organized in plant cells. High level expression of FP-fused tubulin protein causes abnormal plant growth (Abe and Hashimoto 2005). Thus, the unexpected effect of FP-decoration on the state of MTs in plant cells cannot be denied. Transiently expressed FP-tagged G-actin monomers can form filaments in plant cells (Kijima et al. 2018). However, the functionality of G-actin proteins remains unclear in the absence of complementation studies testing whether the expression of FP-fused G-actin can rescue the loss-of-function mutants; therefore, indirect labeling of AFs through FP-fused actin-binding proteins (ABPs), such as Lifeact and Talin, remains the prevailing method to visualize AFs in plant cells (Era et al. 2009; Kost et al. 1998). However, such ABPs may not label all AF types in any plant cell.

Immunostaining can compensate for such drawbacks. It is an antibody-based method allowing to visualize the accumulation and localization of a protein of interest at the cellular level. Immunostaining is still a powerful approach to visualize AFs and MTs, since it does not require generating transgenic reporter lines, and thus is a time-saving method. Moreover, it can also be applied to plant species which cannot be transformed with the fluorescent reporters for MTs and AFs. The organization of AFs and MTs in various tissues of diverse plant species has been unveiled by immunostaining (Ouko et al. 2010; Sun et al. 2017; Takatsuka et al. 2018; Wilsen et al. 2006). Additionally, applying anti-actin and anti-tubulin antibodies produced in different host species allows the simultaneous visualization of AFs and MTs. A previous study has developed a whole-mount immunostaining method using roots of *Arabidopsis thaliana* to simultaneously double-label AFs and MTs, employing primary antibodies raised in rabbit and mouse (Collings and Wasteneys 2005). Here, to make antibodies raised in other host animal species available, such as rat, we examine the specificity of the secondary antibody toward the primary antibody raised in different host-species and propose optimal methods to double-label AFs and MTs, depending on the combinations of the host-species for primary antibodies: “simultaneous immunostaining” and “sequential immunostaining”.

A longitudinal view of *Arabidopsis* root reveals three distinct zones along the differentiation trajectories starting from the tip: the proximal meristem (PM), the transition zone (TZ), and the elongation/differentiation zone (EDZ) (Takatsuka and Umeda 2014). Cells actively divide to increase their number in the PM and start growing in the longitudinal direction after entering the TZ and EDZ, reaching a maximum growth rate in the EDZ. We previously reported that the organization of MTs and AFs are markedly different in the EDZ. Specifically, MTs display a transverse alignment at the cell cortex, while thick AFs are formed in the longitudinal direction (Takatsuka et al. 2018). First, to validate antibody specificity toward antigens in this study, we conducted a single immunofluorescent staining of MTs or AFs and observed their organization in the epidermal EDZ cells. We used commercially available antibodies: two anti-actin antibodies raised in mouse or rabbit (msACT and rbACT, respectively) and one anti-α-tubulin antibody raised in rat (rtTUB). According to our previous study (Takatsuka et al. 2018), whole-mount immunostaining was conducted as follows: (1) four-day-old roots were fixed for 40 min at room temperature in 1 ml PEMT buffer (50 mM PIPES, 2 mM EGTA, 2 mM MgSO_4_·7H_2_O, and 0.05% Triton X-100) containing 1.5% formaldehyde and 0.5% glutaraldehyde in a 1.5 ml tube; (2) after washing with 1 ml of PEMT buffer for three times, cell walls were digested with 0.05% pectolyase Y-23 (Kyowa Chemical, Kagawa, Japan) dissolved in 1 ml PEMT buffer containing 0.4 M mannitol for 20 min at 30°C; (3) after washing with 1 ml of PEMT buffer for three times, samples were dehydrated with 1 ml of chilled methanol for 10 min at −20°C; (4) after washing with 1 ml PBS buffer (130 mM NaCl, 5.1 mM Na_2_HPO_4_, and 1.6 mM KH_2_PO_4_), samples were immersed in 1 ml PBS buffer containing 1 mg ml^−1^ NaBH_4_ for 15 min; (5) after samples were incubated with 1 ml of blocking buffer (PBS buffer containing 50 mM glycine and 1% BSA) for 30 min, they were transferred to a 200 µl tube containing 200 µl of blocking buffer; (6) after removing the blocking buffer, samples were incubated for 16 h at 30°C in 150 µl of blocking buffer containing the primary antibody [monoclonal msACT (Sigma A0605, MA, USA, 1 : 100 dilution), polyclonal rbACT (Agrisera AS13-2640, Vännäs, Sweden, 1 : 100 dilution), and monoclonal rtTUB (Abcam ab6160, Cambridge, UK, 1 : 100 dilution)]; (7) after removing the primary antibody solution, samples were washed with 200 µl PBS buffer containing 50 mM glycine for three times; (8) samples were incubated for 3 h at 37°C in 150 µl of blocking buffer containing the secondary antibody conjugated with fluorophore [goat anti-mouse FITC (Sigma-Aldrich F0257, MA, USA, 1 : 50 dilution), goat anti-rabbit Alexa488 (Thermo Fisher A-31565, MA, USA, 1 : 100 dilution), and goat anti-rat Alexa568 (Abcam ab175476, 1 : 500 dilution)]; (9) after samples were washed with 200 µl PBS for three times, they were put on a 24×60 mm cover glass and a 18×18 mm cover glass was laid on it. (10) observations were made using a confocal laser scanning microscopy (Nikon A1 HD25, Tokyo, Japan) equipped with a 100x silicone immersion objective lens; (11) Z-stack images (approximately 10 µm thick) were taken at 0.5 µm intervals around the cell cortex, and the maximum projection of the Z-stack view was generated using ImageJ software; and (12) colocalization of MTs and AFs in a 5 µm square region at the center of the cell was analyzed using the Image plugin Coloc 2. The Pearson correlation coefficients from 10 PM or EDZ cells were calculated and represented as mean±SD.

The observations of single MT or AF immunofluorescent staining with the primary antibody and the intended secondary antibodies (msACT & anti-mouse secondary, rbACT & anti-rabbit secondary, and rtTUB & anti-rat secondary) indicated transversely aligned MTs and longitudinally aligned AFs in the epidermal EDZ, respectively, consistent with our previous study (Takatsuka et al. 2018) (Supplementary Figure S1A–C; left panels). These results indicate that the primary and secondary antibodies used properly recognize their antigen. Then, we tested whether cross-species binding occurs between the primary and the secondary antibodies produced in different host species. The combination of rtTUB & anti-mouse secondary displayed faint yet obvious MT-like structures in the channel for anti-mouse secondary, indicative of a cross-species binding of the anti-mouse secondary to rtTUB (Supplementary Figure S1C; middle panel). In contrast, the anti-rat secondary antibody exhibited scarcely detectable cross-species binding to msACT (Supplementary Figure S1A; right panel). These results are consistent with a previous study on immunostaining with animal cells that showed anti-mouse secondary cross-reacted with anti-rat primary, whereas anti-rat secondary did not show affinity to anti-mouse primary (Mao et al. 2021). No obvious cross-species binding was detected between the antibodies raised in rabbit and rat (Supplementary Figure S1B, C; right panels).

Next, we proceeded to simultaneous double immunolabeling of AFs and MTs. msACT or rbACT were applied with rtTUB to the root samples, followed by the application of the corresponding secondary antibodies (Figure 1A). At low magnification, no striking differences were observed in the staining patterns between msACT & rtTUB and rbACT & rtTUB; strong fluorescent signals were detected from channels for green and red signals (Figure 1B). However, a closer look at the EDZ cells of msACT & rtTUB revealed transversely aligned MT-like structures in the green channel intended for msACT (Figure 1C, D). Quantitative analysis showed a high degree of colocalization of the signals from the channels intended for msACT and rtTUB (Figure 1E), indicating that simultaneous double-immunostaining combining msACT & rtTUB is practically unfeasible due to a cross-species binding of the anti-mouse secondary to rtTUB, as observed in Supplementary Figure S1C. By contrast, in the EDZ cells simultaneously stained with rbACT and rtTUB, undesirable MT- and AF-like structures were not detected in the channels for anti-mouse FITC (green) and anti-rat Alexa568 (red) secondaries, respectively (Figure 1C–E). Similarly, in the basal PM cells, no obvious MT and AF colocalization was observed, even though they formed similar mesh-like structures (Figure 1C–E), indicative of a negligible level of cross-species binding between rbACT and anti-rat secondary and between rtTUB and anti-rabbit secondary. The “simultaneous whole-mount immunostaining” with rtTUB and rbACT provides a method to visualize AFs and MTs simultaneously in one sample.

**Figure 1:**
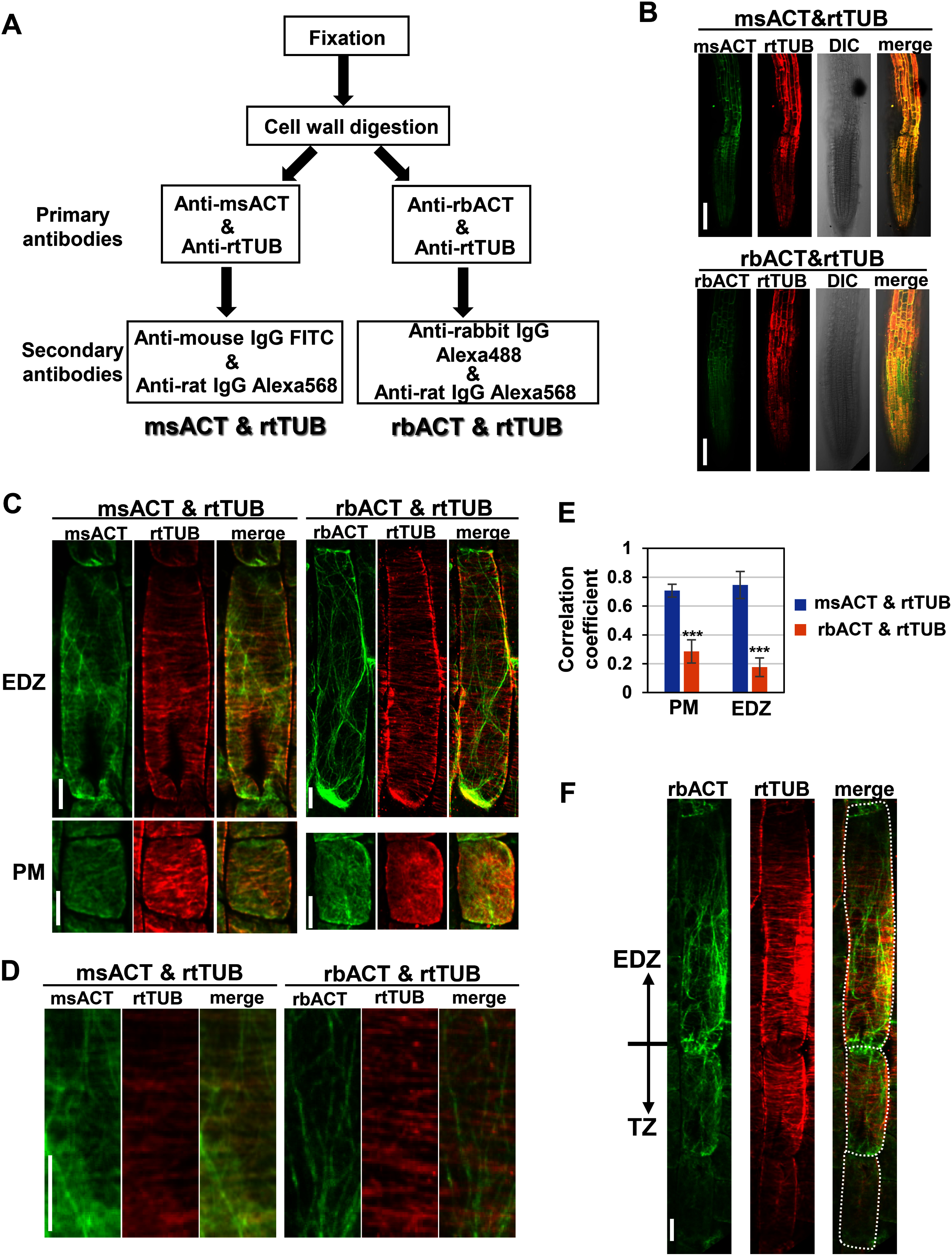
Figure 1. Simultaneous whole-mount immunostaining using rat anti-α-tubulin and mouse anti-actin or rabbit anti-actin primary antibodies. (A) A schematic diagram of the simultaneous whole-mount immunostaining method, in which two sets of primary and secondary antibodies are applied simultaneously. (B) Root tips of five-day-old wild-type seedlings stained with mouse anti-actin and rat anti-tubulin (msACT & rtTUB) or rabbit anti-actin and rat anti-tubulin (rbACT & rtTUB), visualized with the fluorophore-conjugated secondary antibodies. Scale bars, 100 µm. (C) Images of the immunostained cells in the basal region of the proximal meristem (PM) and in the elongation/differentiation zone (EDZ). Scale bars, 10 µm. (D) Magnified images of the EDZ cells shown in C. Scale bar, 10 µm. (E) Colocalization analysis of the signals from the channels intended for MTs and AFs in the PM or EDZ cells, using the Pearson correlation coefficient. Data are presented as mean±SD (*n*=10). Significant differences were determined using Student’s *t*-test *** *p*<0.0001. (F) Images of the last two transition zone (TZ) and the first EDZ cells subjected to simultaneous double-immunostaining with rbACT & rtTUB. White dotted lines in the right panel indicate the cell outlines. Scale bar, 10 µm.

Next, we examined MT and AF organization at the boundary between the TZ and EDZ, determined by a cell 1.5-fold longer than the abutting lower last TZ cell, as defined in our previous study (Takatsuka et al. 2018). The results indicated that AFs were rearranged from the fine, meshed network to the longitudinally oriented, thick bundles during the transition from the TZ to the EDZ (Figure 1F). In contrast, the alignment of MTs into the transverse direction was completed before entering the EDZ (Figure 1F). These results are consistent with MT and AF observations made separately using a single immunofluorescence method (Takatsuka et al. 2018).

As mentioned above, our attempt for simultaneous whole-mount immunostaining of AFs and MTs using msACT and rtTUB failed because of a cross-species binding of the secondary antibodies (Figure 1C–E). A previous study in the animal field has demonstrated that such a cross-species binding could be overcome by a step-by-step method, in which two rounds of reaction were conducted to immunolabel two antigens separately (Mao et al. 2021). Following this method, we conducted sequential double-immunostaining with msACT and rtTUB (Figure 2A). Before the second round of reaction, excessive free secondary antibody applied in the first round was carefully washed away with PBS buffer to inhibit its binding to the primary antibody of the second round. Sequential staining starting from rtTUB and anti-rat secondary, followed by msACT and anti-mouse secondary (“rtTUB→msACT”) displayed MT-like transversely aligned filaments in the green channel intended to detect msACT (Figure 2B–D). The undesirable colocalization of the MT and AF signals was quantitatively shown in Figure 2E. This result is likely due to a cross-species binding between the anti-mouse secondary and rtTUB that was unbound to the anti-rat secondary in the first round. In contrast, msACT and anti-mouse secondary followed by rtTUB and anti-rat secondary (“msACT→rtTUB”) displayed MT-like structures in the channel for anti-rat Alexa568 but not for anti-mouse FITC (Figure 2C–E). This result indicates that a cross-species binding of the secondary produced in rat to msACT was below detectable levels under our microscopy settings, consistent with the finding that anti-rat secondary exhibited a negligible level of affinity toward the primary raised in mouse (Supplementary Figure S1A). With this protocol, changes in the organization of AFs and MTs during the TZ to EDZ transition were successfully visualized in the same sample (Figure 2F). Our “sequential whole-mount immunostaining” using msACT and rtTUB provides another method for simultaneously visualizing AFs and MTs and allows for a wider choice of antibodies. Furthermore, future development of our method will enable “triple-labeling”, which offers a powerful tool to examine whether and how a protein of interest, whose antibody is raised in rabbit, is associated with AFs and MTs, labeled by msACT and rtTUB, respectively. In addition, the findings presented here may provide useful information for selecting antibodies when double-immunostaining is conducted for proteins other than actin and tubulin.

**Figure 2:**
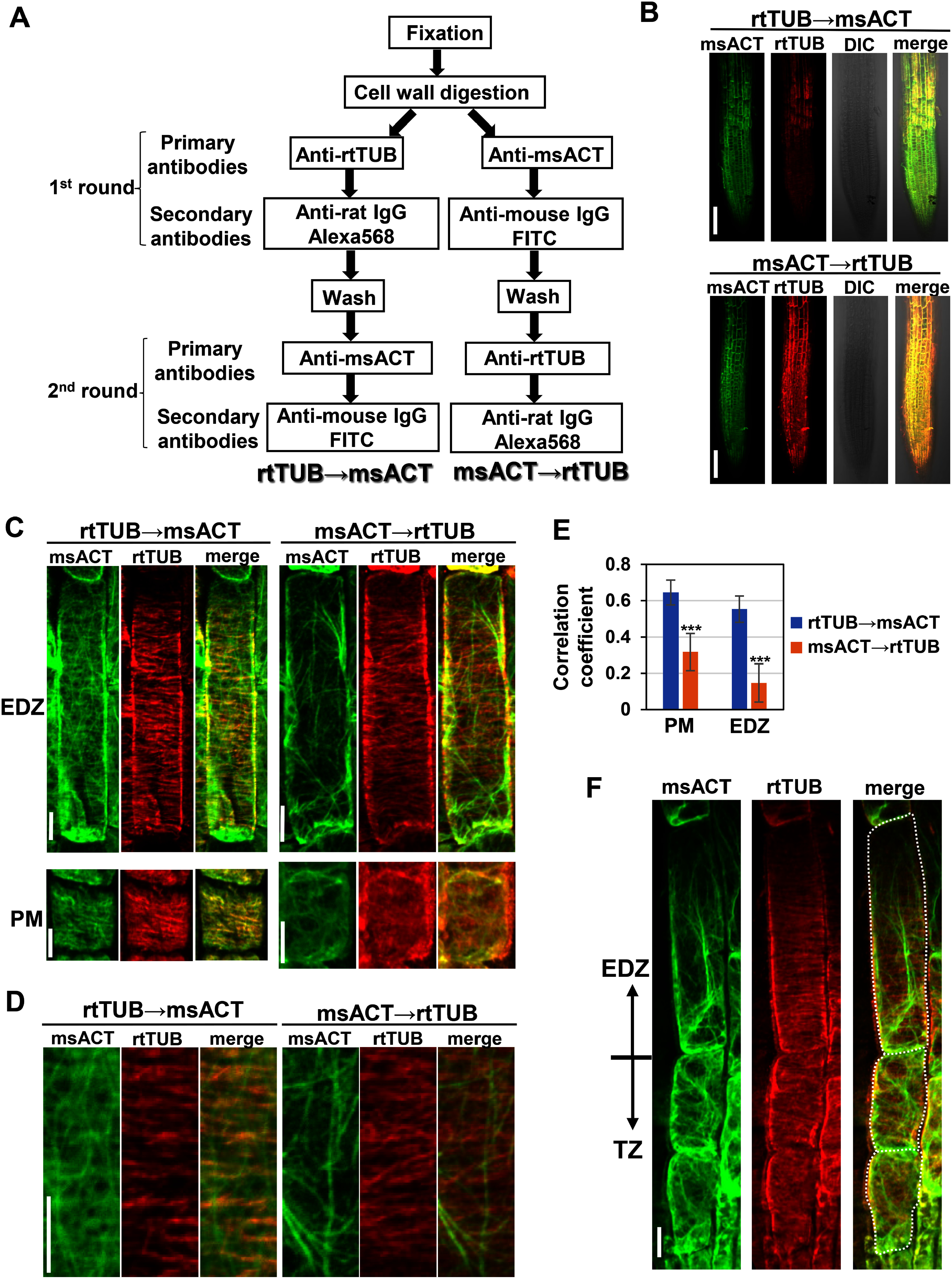
Figure 2. Sequential whole-mount immunostaining using rat anti-α-tubulin and mouse anti-actin primary antibodies. (A) A schematic diagram of the sequential double-immunostaining method, in which two rounds of reactions are conducted sequentially to immunolabel two antigens separately. (B) Root tips of five-day-old wild-type seedlings stained with rat anti-tubulin, followed by mouse anti-actin (rtTUB→msACT) or mouse anti-actin, followed by rat anti-tubulin (msACT→rtTUB). Scale bars, 100 µm. (C) Images of immunostained cells in the basal region of the PM and in the EDZ. Scale bars, 10 µm. (D) Magnified images of the EDZ cells shown in C. Scale bar, 10 µm. (E) Colocalization analysis of the signals from channels intended for MTs and AFs in the PM or EDZ cells, using the Pearson correlation coefficient. Data are presented as mean±SD (*n*=10). Significant differences were determined using Student’s *t*-test *** *p*<0.0001. (F) Images of the last two TZ and first EDZ cells subjected to simultaneous double-immunostaining with msACT→rtTUB. White dotted lines in the right panel indicate the cell outlines. Scale bar, 10 µm.

This study proposes a highly useful method for simultaneous visualization of MTs and AFs. It should be noted, however, that the method proposed in this study is optimized for *Arabidopsis* root epidermal cells and that it has not yet been determined if it can be applied to other plant species and cell types. Future studies will explore optimal conditions for fixation, cell wall digestion, and/or antibody concentration for plant species and cell types of interest, which will further extend the usefulness of our methods.

## Abbreviations

AF: actin filament
EDZ: elongation/differentiation zone
msACT: mouse anti-actin antibody
MT: microtubule
PM: proximal meristem
rbACT: rabbit anti-actin antibody
rtTUB: rat anti-tubulin antibody
TZ: transition zone

## Description of Supplementary Files

Supplementary Figure S1. Single immunofluorescent staining to validate antibody specificity.
