# Supplementary material for: Whole-mount immunostaining that avoids cross-reaction between antibodies from different host species for simultaneous visualization of actin filaments and microtubules
Source: Plant Biotechnol (Tokyo). 2025 Mar 25;42(1):87–92. doi: 10.5511/plantbiotechnology.24.1103a (PMC12622907; doi:10.5511/plantbiotechnology.24.1103a)
Supplement: Supplementary Data [file plantbiotechnology-42-1-24.1103a-s001.pdf]

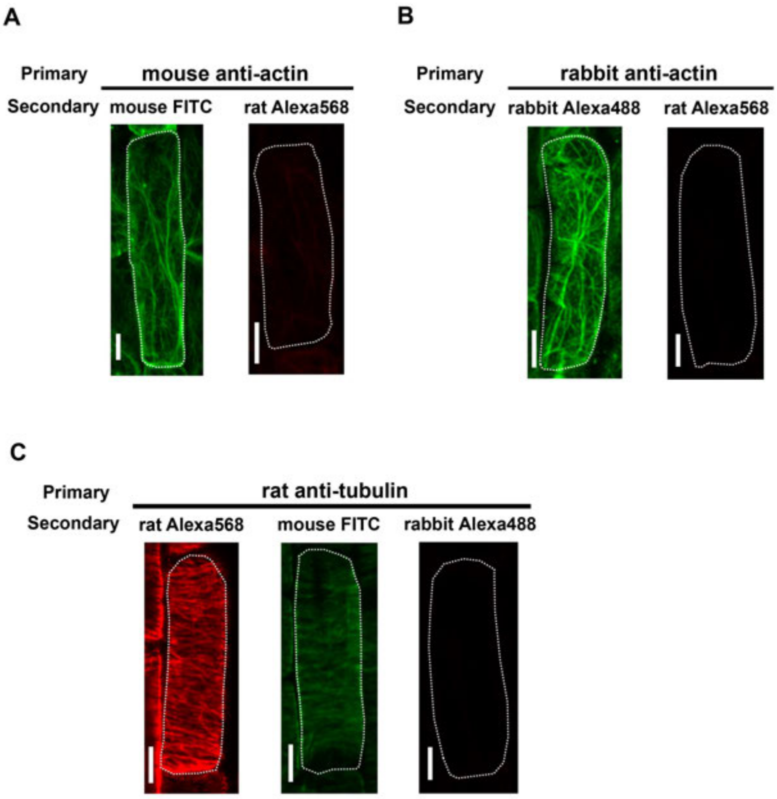

Supplementary Figure S1. Single immunofluorescent staining to validate antibody specificity.
